# Short-term residential exposure to air pollution and risk of acute myocardial infarction deaths at home in China

**DOI:** 10.1007/s11356-023-27813-5

**Published:** 2023-05-29

**Authors:** Jian Cheng, Hao Zheng, Jing Wei, Cunrui Huang, Hung Chak Ho, Shengzhi Sun, Dung Phung, Ho Kim, Xiling Wang, Zhongliang Bai, Mohammad Zahid Hossain, Shilu Tong, Hong Su, Zhiwei Xu

**Affiliations:** 1 Department of Epidemiology and Biostatistics, School of Public Health, Anhui Medical University, Hefei, China; 2 Anhui Province Key Laboratory of Major Autoimmune Disease, Hefei, China; 3 Department of Environmental Health, Jiangsu Provincial Center for Disease Control and Prevention, Nanjing, China; 4 Department of Atmospheric and Oceanic Science, Earth System Science Interdisciplinary Center, University of Maryland, College Park, MD USA; 5 Vanke School of Public Health, Tsinghua University, Beijing, China; 6 Department of Public and International Affairs, City University of Hong Kong , Hong Kong, China; 7 School of Public Health, Capital Medical University, Beijing, China; 8 School of Public Health, Faculty of Medicine, University of Queensland, Brisbane, Australia; 9 Department of Public Health Science, Graduate School of Public Health, Seoul National University, Seoul, Republic of Korea; 10 Institute of Health and Environment and Graduate School of Public Health, Seoul National University, Seoul, Republic of Korea; 11 School of Public Health, Fudan University, Shanghai, China; 12 Shanghai Key Laboratory of Meteorology and Health, Shanghai Meteorological Service, Shanghai, China; 13 School of Health Services Management, Anhui Medical University, Hefei, China; 14 International Centre for Diarrhoeal Disease Research, Bangladesh (icddr,b), Dhaka, Bangladesh; 15 Department of Clinical Epidemiology and Biostatistics, Shanghai Children’s Medical Center, Shanghai Jiaotong University School of Medicine, Shanghai, China; 16 School of Public Health, Institute of Environment and Population Health, Anhui Medical University, Hefei, China; 17 Center for Global Health, Nanjing Medical University, Nanjing, China; 18 School of Public Health and Social Work, Queensland University of Technology, Brisbane, Australia; 19 School of Medicine and Dentistry, Griffith University, Gold Coast, Queensland 4222 Australia

**Keywords:** Air pollution, Particulate matter, Gaseous pollutant, Acute myocardial infarction, Cardiovascular disease

## Abstract

Air pollution remains a major threat to cardiovascular health and most acute myocardial infarction (AMI) deaths occur at home. However, currently established knowledge on the deleterious effect of air pollution on AMI has been limited to routinely monitored air pollutants and overlooked the place of death. In this study, we examined the association between short-term residential exposure to China’s routinely monitored and unmonitored air pollutants and the risk of AMI deaths at home. A time-stratified case-crossover analysis was undertaken to associate short-term residential exposure to air pollution with 0.1 million AMI deaths at home in Jiangsu Province (China) during 2016–2019. Individual-level residential exposure to five unmonitored and monitored air pollutants including PM_1_ (particulate matter with an aerodynamic diameter ≤ 1 μm) and PM_2.5_ (particulate matter with an aerodynamic diameter ≤ 2.5 μm), SO_2_ (sulfur dioxide), NO_2_ (nitrogen dioxide), and O_3_ (ozone) was estimated from satellite remote sensing and machine learning technique. We found that exposure to five air pollutants, even below the recently released stricter air quality standards of the World Health Organization (WHO), was all associated with increased odds of AMI deaths at home. The odds of AMI deaths increased by 20% (95% confidence interval: 8 to 33%), 22% (12 to 33%), 14% (2 to 27%), 13% (3 to 25%), and 7% (3 to 12%) for an interquartile range increase in PM_1_, PM_2.5_, SO_2_, NO_2_, and O_3_, respectively. A greater magnitude of association between NO_2_ or O_3_ and AMI deaths was observed in females and in the warm season. The greatest association between PM_1_ and AMI deaths was found in individuals aged ≤ 64 years. This study for the first time suggests that residential exposure to routinely monitored and unmonitored air pollutants, even below the newest WHO air quality standards, is still associated with higher odds of AMI deaths at home. Future studies are warranted to understand the biological mechanisms behind the triggering of AMI deaths by air pollution exposure, to develop intervention strategies to reduce AMI deaths triggered by air pollution exposure, and to evaluate the cost-effectiveness, accessibility, and sustainability of these intervention strategies.

**Graphical abstract:**

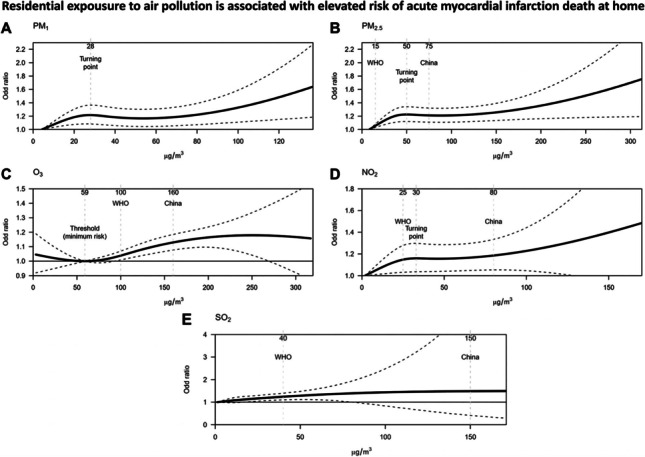

**Supplementary Information:**

The online version contains supplementary material available at 10.1007/s11356-023-27813-5.

## Introduction

Air pollution is a widely recognized and inhalable threat to cardiovascular health (GBD 2019 Risk Factors Collaborators 2020; Rajagopalan et al. 2018). As one of the major contributors to cardiovascular morbidity and mortality, acute myocardial infarction (AMI) attacks remain a major public health problem in China, leading to massive burden of hospitalizations and deaths in the past decades (Chang et al. 2017; Liu et al. 2017; Li et al. 2022). There has been an increasing number of studies reporting an increased risk of AMI attacks within few days after exposure to particulate or gaseous air pollutants such as PM_2.5_ (particulate matter with an aerodynamic diameter ≤ 2.5 μm), PM_10_ (particulate matter with an aerodynamic diameter ≤ 10 μm), SO_2_ (sulfur dioxide), NO_2_ (nitrogen dioxide), and O_3_ (ozone) (Rajagopalan et al. 2018; Claeys et al. 2016; Liu et al. 2021). Aside from these routinely monitored and widely studied air pollutants in many regions of the world, many routinely unmonitored air pollutants such as PM_1_ (particulate matter with an aerodynamic diameter ≤ 1 μm) have been increasingly reported to have adverse and greater health impacts (Chen et al., 2017a, b; Hu et al. 2018; Yen and Chen 2022). However, the effect of PM_1_ on AMI deaths remains largely unknown so far (Mei et al. 2022; Xu et al. 2022; Song et al. 2022).

AMI attacks have marked circadian rhythm, with incidents occurring more during midnight or early morning (Jia et al. 2012; Mohammad et al. 2018; Sert Kuniyoshi et al. 2008). AMI attacks and deaths often happen when patients are at home (Cross and Warraich 2019; Wu et al. 2021). However, the existing studies investigating the association between short-term exposure to air pollution and risk of AMI deaths mainly used city-level air pollution data, which was subject to exposure measurement bias (Bhaskaran et al. 2011; Liang et al. 2018). One recent study in China utilized air pollution data from ground monitoring stations and applied geographic prediction model to derive spatially resolved air pollutant data (Liu et al. 2021), but the prediction model had a less ideal performance for some pollutants such as SO_2_ (*R* square was 0.58). In addition, most previous studies on air pollution and AMI deaths estimated air pollution exposure based on a limited number of ground air quality monitoring stations (Chen et al. 2021; Yu et al. 2018; Zhu et al. 2017), failing to consider the air pollution level within the residential areas of decedents.

The recent advances in using satellite remote sensing and spatiotemporal models to derive air pollution data with high spatial resolution present opportunities to reduce exposure measurement bias in assessing the health impacts of air pollution. In this study, we utilized high-resolution air pollution data and conducted a multi-region time-stratified case-crossover analysis to investigate if short-term residential exposure to air pollutants (PM_1_, PM_2.5_, NO_2_, O_3_, and SO_2_) was associated with risk of AMI deaths at home in China. Furthermore, we attempted to examine if sex, age, educational attainment, and season modified the association between air pollutants and AMI deaths.

## Methods

### Study area and data collection

This study was conducted in Jiangsu Province, China. Jiangsu is an eastern coastal province of China and had 77 million inhabitants in its 13 cities in 2016. Previous studies have reported an association between exposure to air pollutants and the risk of cardiovascular mortality in some cities of Jiangsu Province (Chen et al. 2021; Yu et al. 2018; Zhu et al. 2017; Song et al. 2022), although they largely used city-level air pollution exposure as an approximation of individual-level air pollution exposure. We chose Jiangsu Province as the research site also because of our established collaborations with the local government agency (i.e., Jiangsu Provincial Center for Disease Control and Prevention).

Data on deaths from AMI (International Classification of Diseases, tenth version: I21) in these 13 cities from January 1, 2016 to 31 December 2019 were obtained from Jiangsu Provincial Centre for Disease Control and Prevention. For each decedent, we extracted information on their residential address, place of death, date of death, age, sex, and educational attainment. Those deaths which occurred at home were identified from the “place of death” variable and were included in this study. Age at death was categorized into four groups, including < 65, 65–74, 75–84, and ≥ 85 years. Educational attainment was dichotomized as “< 10 years” or “≥ 10 years.” We only included AMI deaths that occurred in the study region and within the study period, and we excluded those whose individual information (e.g., age, sex) was incomplete or not available.

For exposure data, we obtained satellite-derived air pollution data including daily concentrations of PM_1_, PM_2.5_, NO_2_, SO_2_, and O_3_. Briefly, daily air pollutant concentrations were predicted across China using a series of models with a machine learning technique called “space–time extremely randomized trees.” These models used air pollution data from ground-monitoring network, satellite remote sensing products (National Aeronautics and Space Administration’s (NASA) Terra and Aqua MODIS aerosol products), and other variables (e.g., weather conditions and land cover) as data input, and predicted daily concentrations of air pollutants at a high spatial resolution. The spatial resolutions of data on PM_1_ and PM_2.5_ and data on NO_2_, SO_2_, and O_3_ were 1 km and 10 km, respectively. Results from the 10-fold cross-validations suggested a high prediction accuracy for each pollutant (*R* square was 0.77 for PM_1_, 0.90 for PM_2.5_, 0.84 for NO_2_, 0.84 for SO_2_, and 0.87 for O_3_). The details of the air pollution data have been previously described (Wei et al. 2019, 2020, 2021, 2022). To adjust for temperature in the model assessing the association between air pollution and AMI deaths, we also collected hourly air temperature data from an enhanced global dataset for “the land component of the fifth generation of European reanalysis” (ERA5-land), and this air temperature data had a spatial resolution of 9 km (CDS 2021; Hersbach et al. 2020). Daily mean temperature values were calculated by averaging hourly air temperatures within 24 h. To measure residential exposure to air pollution and temperature, each decedent’s residential address was geocoded and then matched to daily spatially gridded values.

### Study design

We used a time-stratified case-crossover study design to quantify the association between short-term exposure to air pollutants and risk of AMI deaths. Specifically, for each decedent, the date of death was the “case” day and “control” days were the same days in the weeks before and after the death within the same month and calendar year. For instance, if one AMI case died on Sunday, December 21, 2018 (i.e., “case” day), “control” days would have been all other Sundays within this month, including December 7, 14, and 28, 2018. This study design was chosen because it has two merits. Firstly, it can examine the association of AMI death risk with air pollution exposure at individual level. Secondly, it can automatically adjust for long-term trend and seasonality of health outcome as well as time-invariant individual variables such as age, sex, and cigarette smoking (Bhaskaran et al. 2011).

### Statistical analyses

A conditional logistic regression model was utilized to fit the association between air pollution exposure and AMI death risk. To account for the temporal auto-correlation of daily air pollutant concentrations and capture the potential nonlinear association between air pollution exposure and AMI death risk (Yan et al. 2019), we used the logistic regression model combined with a distributed lag nonlinear model (DLNM). For each air pollutant, we used a cross-basis function in DLNM to simultaneously capture exposure-response and lag-response associations. A natural cubic spline function with three degrees of freedom and a maximum lag of 7 days were used for each pollutant. We also included daily mean temperature as a covariate in the models, and a natural cubic spline function with three degrees of freedom and a maximum lag of 14 days were used for temperature. The used model was as follows:

$$\textrm{logit}\left(P\left(\textrm{case}=1\ \textrm{in}\ {\textrm{stratum}}_{\textrm{i}}\ |\ \textrm{air}\ \textrm{pollutant},\textrm{covariate}\right)\right)={\beta}_{0,{\textrm{stratum}}_{\textrm{i}}}+{\beta}_1\ast \textrm{air}\ \textrm{pollutant}+\beta \ast \textrm{covariate}$$

where stratum_i_ refers to the fixed time strata *i* (i.e., the matched case and control periods in the same calendar month in the same year); ${\beta}_{0,{\textrm{stratum}}_{\textrm{i}}}$indicates the intercept of stratum *i*; *β*_1_ ∗ air pollutant refers to the estimated coefficient *β*_1_ for each air pollutant; *β* ∗ covariate is the coefficient *β* for mean temperature.

Although the regression model with a linear or piecewise linear function may perform better statistically, we used a nonlinear function in model construction because it is closer to the complex nature of exposure-response association (Yan et al. 2019). This modeling strategy was chosen in alignment with previous studies (Liu et al. 2021; Bhaskaran et al. 2011), and based on the Bayesian information criterion. Stratified analyses were conducted to examine whether age, sex, educational attainment, and season (cold season: October to March; warm season: April to September) modified the association between air pollution exposure and AMI death risk. Result comparisons between dichotomized subgroups were examined through a two-sample *z*-test and trend test for ordinal categorical subgroups was performed by meta-regression technique.

All data analyses were performed using packages “survival” and “dlnm” in R software version 4.1.0. A two-sided *P*-value < 0.05 was considered statistically significant. We estimated odds ratio (OR) and its 95% confidence interval (CI) at a single lag day and accumulated over few days associated with an interquartile range (IQR) increase in air pollutant concentration (Guo et al. 2013). Besides, we estimated the potential minimum risk threshold and risk turning point of air pollutant from the above-fitted nonlinear exposure-response associations. Minimum risk threshold refers to a specific concentration value above which death risk increases continuously and risk turning point refers to a specific concentration above which death risk increment pattern starts to change (Chen et al., 2017a, b; Li et al. 2021). To quantify the association of AMI death risk with exposure to air pollutant concentrations below the air quality standards recommended by China and the World Health Organization (WHO), we compared the minimum risk threshold with the thresholds recommended in the China and WHO air quality standards.

### Sensitivity analysis

Two sensitivity analyses were conducted. First, in recognition of the fact that people who died from AMI may not always stay indoors prior to death, we estimated the air pollution exposure within different spatial buffer areas (i.e., the radii of 10 km and 20 km within their residential address) and examined the association of AMI death risk with air pollution exposure within these buffer areas. Second, aside from one-pollutant models used for the main analysis, we also used two-pollutant models.

## Results

### Characteristics of the study population and exposures

There were 102,183 AMI deaths at home during the study period (Supplementary Fig. S1), and 49.3% of them were males (Supplementary Table S1). The mean death age was 78.8 years and 85.8% of the deaths occurred in elderly people (≥ 65 years). Most of these decedents had ≥ 10 years of education (97.0%) and more than half of the deaths occurred in the cold season (57.3%).

The mean values of PM_1_, PM_2.5_, NO_2_, SO_2_, and O_3_ on case days were 33.6 μg/m^3^, 55.3 μg/m^3^, 35.2 μg/m^3^, 16.6 μg/m^3^, and 102.6 μg/m^3^, respectively (Table 1). The correlation coefficients between two pollutants included in two-pollutant models were all less than 0.6 and less than 0.5 in most cases. The mean values of all air pollutants (except for NO_2_) on case days were higher than that on control days (*P* < 0.05). Meanwhile, during the days when AMI deaths happened, there were 20.4%, 0.9%, 0%, and 10.6% of days exceeding the Chinese air quality standards for PM_2.5_, NO_2_, SO_2_, and O_3_, respectively. During the days when AMI deaths happened, there were 99.5%, 71.0%, 2.7%, and 43.9% of days exceeding the WHO air standards for PM_2.5_, NO_2_, SO_2_, and O_3_, respectively.

**Table 1:** Descriptive statistics of air pollutant concentration and mean temperature in Jiangsu province of China, 2016–2019

Variables	IQR	Exposure on case days			Exposure on control days			Percentage of “case days” exceeding air quality criterion	
		Mean	Median	Range	Mean	Median	Range	China	Word Health Organization (WHO)
PM_1_ (μg/m^3^)	17.9	33.6	30.4*	4.6 to 133.5	33.5	30.3	4.3 to 136.6	NA	NA
PM_2.5_ (μg/m^3^)	34.9	55.3	48.0*	9.1 to 283.5	54.9	47.5	8.3 to 312.6	20.4%	99.5%
NO_2_ (μg/m^3^)	19.1	35.2	32.4	2.7 to 170.1	35.1	32.4	1.9 to 158.1	0.9%	71.0%
SO_2_ (μg/m^3^)	10.4	16.6	14.3*	1.3 to 171.1	16.5	14.2	1.2 to 171.1	0%	2.7%
O_3_ (μg/m^3^)	61.3	102.6	94.1*	3.5 to 301.1	102.2	93.8	3.1 to 317.1	10.6%	43.9%
Temperature (°C)	17.3	14.3	14.2*	-9.9 to 34.1	14.4	14.4	-9.6 to 34.3	-	-

IQR is interquantile range; differences between case and control days in air pollutant concentrations and mean temperature were tested using Wilcoxon rank test, with * referring to *P* < 0.05; air quality standards for PM_2.5_ are 75 μg/m^3^ (China) and 15 μg/m^3^ (WHO), for NO_2_ are 80 μg/m^3^ (China) and 25 μg/m^3^ (WHO), for SO_2_ are 150 μg/m^3^ (China) and 40 μg/m^3^ (WHO), for O_3_ are 160 μg/m^3^ (China) and 100 μg/m^3^ (WHO); NA means no existing air quality standard for that air pollutant

## Association between short-term exposure to air pollutants and risk of AMI deaths

**Fig. 1:**
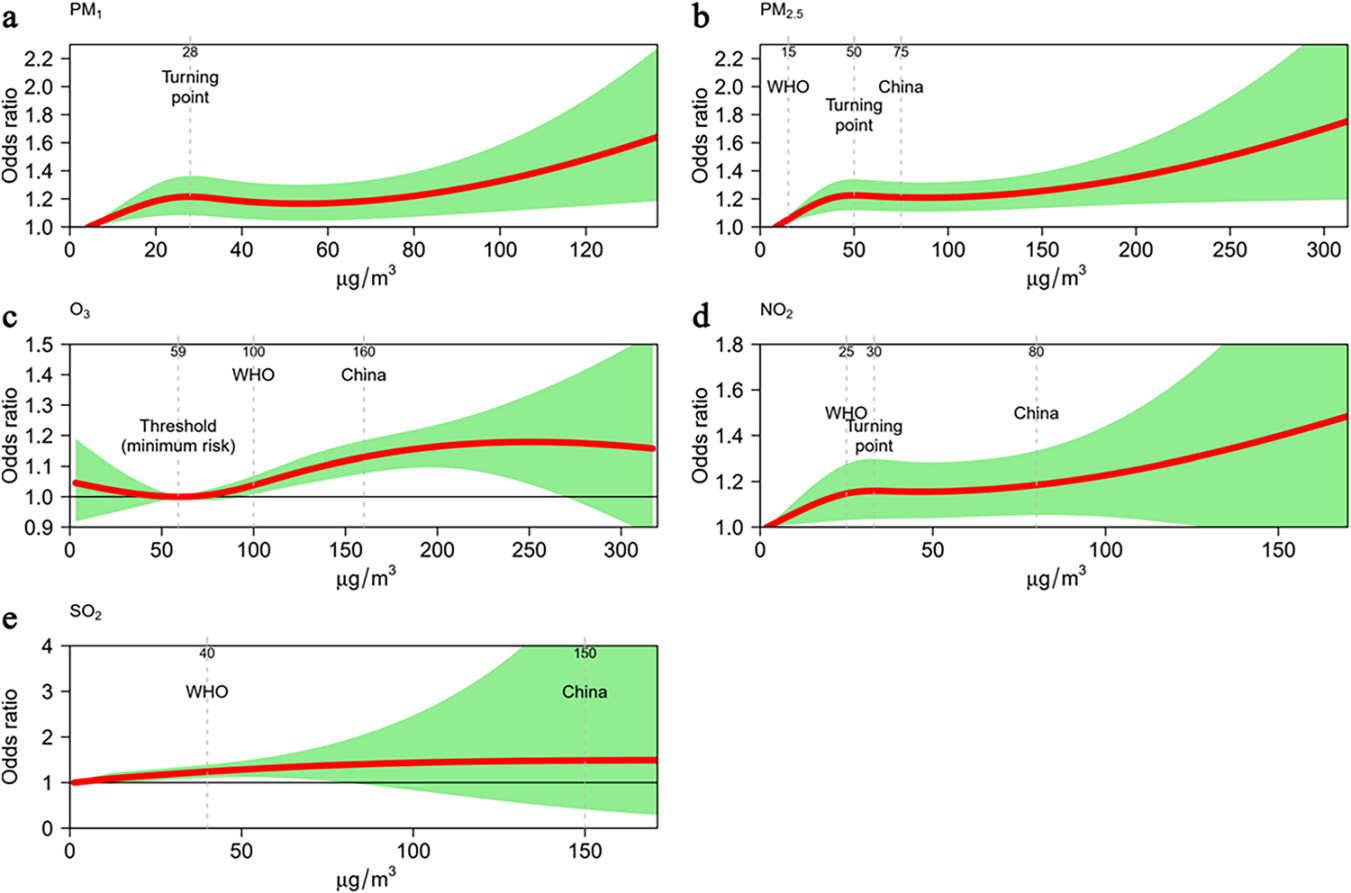
Exposure-response association between exposure to air pollutants and odds of AMI deaths at home. Solid red line is the estimated point effect of air pollutant and shaded green area is the 95% confidence interval; exposure-response association curves were plotted with lag0–1 for PM_1_, lag0–1 for PM_2.5_, lag0–2 for NO_2_, lag0–5 for SO_2_, and lag0–5 for O_3_

Figure 1 shows the exposure-response association between exposure to five air pollutants and odds of AMI deaths at home. For PM_1_, PM_2.5_, and NO_2_, odds of AMI deaths increased sharply when people were exposed to these air pollutants under certain level (i.e., a turning point). The identified turning points for PM_1_, PM_2.5_, and NO_2_ were 28 μg/m^3^ (lag0–1), 50 μg/m^3^ (lag0–1), and 30 μg/m^3^ (lag0–2), respectively. For O_3_, odds of AMI deaths started to increase when O_3_ concentration exceeded 59 μg/m^3^ (lag0–5). There was a monotonical increase in odds of AMI deaths after exposure to SO_2_ (lag0–2). Noticeably, for all air pollutants included in the China or WHO air quality standards, exposure to concentrations lower than the WHO or China air quality standards was still associated with higher odds of AMI deaths.

The specific effect estimates for the association between air pollutants and odds of AMI deaths are shown in Table 2. For each air pollutant, the greatest value of effect estimates across different lags is presented. The odds of AMI deaths increased by 20% (95%CI: 8 to 33%), 22% (95%CI: 12 to 33%), 13% (95%CI: 3 to 25%), 14% (95%CI: 2 to 27%), and 7% (95%CI: 3 to 12%) for an IQR increase in PM_1_, PM_2.5_, NO_2_, SO_2_, and O_3_, respectively.

**Table 2:** Association between exposure to air pollutants and odds of acute myocardial infarction deaths at home

Pollutants	Odds ratio (95% confidence interval)		
	IQR increase	China’s air quality criteria	WHO’s air quality criteria
PM_1_	1.20 (1.08, 1.33)	NA	NA
PM_2.5_	1.22 (1.12, 1.33)	1.21 (1.11, 1.32)	1.05 (1.03, 1.08)
NO_2_	1.13 (1.03, 1.25)	1.19 (1.05, 1.33)	1.15 (1.03, 1.28)
SO_2_	1.14 (1.02, 1.27)	1.49 (0.41, 5.37)	1.24 (1.10, 1.40)
O_3_	1.07 (1.03, 1.12)	1.13 (1.08, 1.18)	1.04 (1.01, 1.07)

The effect estimates presented in this table are the highest values across different lags. IQR is interquartile range; air quality standards for PM_2.5_ are 75 μg/m^3^ (China) and 15 μg/m^3^ (WHO), for NO_2_ are 80 μg/m^3^ (China) and 25 μg/m^3^ (WHO), for SO_2_ are 150 μg/m^3^ (China) and 40 μg/m^3^ (WHO), for O_3_ are 160 μg/m^3^ (China) and 100 μg/m^3^ (WHO); NA means no existing air quality standard for that air pollutant

**Fig. 2:**
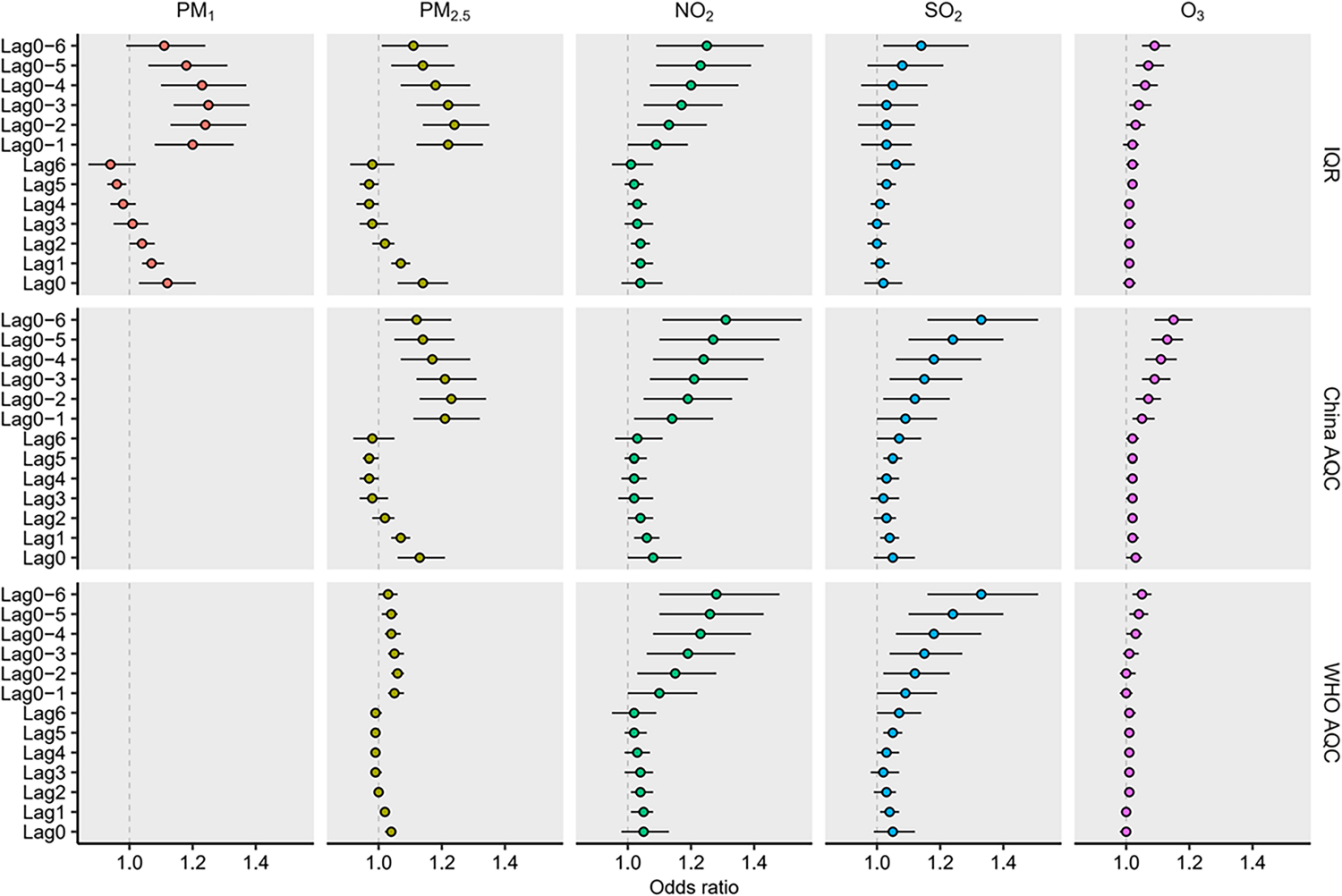
Association between short-term exposure to air pollution and odds of acute myocardial infarction deaths at home across different lags. AQC, air quality criterion; WHO, World Health Organization

The timing when odds of AMI deaths increased significantly after exposure to air pollutants is presented in Fig. 2. When exposed to PM_1_ or PM_2.5_, odds of AMI deaths increased significantly on the same day of exposure or one day after exposure. Specifically, PM_1_- and PM_2.5_-related OR estimates ranged from 1.12 at lag0 to 0.94 at lag6 and from 1.14 at lag0 to 0.98 at lag6, respectively. When exposed to NO_2_, SO_2_, or O_3_, odds of AMI deaths increased a few days after exposure, with OR estimates ranging from 1.04 at lag1 to 1.01 at lag6, from 1.06 at lag6 to 1.00 at lag3, and from 1.02 at lag 6 to 1.01 at lag0, respectively.

### Modification effect of age, sex, educational attainment, and season

Effect estimates for the association between exposure to air pollutants and odds of AMI deaths in different subgroups are shown in Table 3. A greater magnitude of association between exposure to NO_2_ or O_3_ and increased odds of AMI deaths was observed in females and in the warm season. The greatest association between PM_1_ exposure and increased odds of AMI deaths was found in individuals aged ≤ 64 years (OR: 1.31, 95%CI: 1.15 to 1.50), and the magnitude of this association decreased with the increase of age (*P-*value = 0.03).

**Table 3:** Odds of acute myocardial infarction deaths at home for an interquartile range (IQR) increase in air pollutants

Subgroups	Odds ratio (95% confidence interval)				
	PM_1_	PM_2.5_	NO_2_	SO_2_	O_3_
Males	1.28 (1.11, 1.49)	1.25 (1.10, 1.42)	1.00 (0.87, 1.15)	1.05 (0.90, 1.22)	1.01 (0.96, 1.07)
Females	1.12 (0.97, 1.30)	1.19 (1.05, 1.35)	1.27 (1.11, 1.46)	1.12 (0.97, 1.30)	1.13 (1.07, 1.19)
Gender difference	*P* = 0.21	*P* = 0.59	*P* = 0.02*	*P* = 0.55	*P* < 0.01*
≤ 64 years	1.31 (1.15, 1.50)	1.17 (1.04, 1.31)	1.01 (0.89, 1.15)	0.99 (0.86, 1.13)	0.98 (0.93, 1.03)
65–74 years	1.30 (1.15, 1.47)	1.42 (1.28, 1.58)	1.20 (1.07, 1.35)	1.28 (1.13, 1.46)	1.13 (1.08, 1.19)
75–84 years	1.18 (1.08, 1.28)	1.20 (1.12, 1.29)	1.12 (1.03, 1.21)	1.03 (0.94, 1.12)	1.06 (1.03, 1.09)
≥ 85 years	1.14 (1.06, 1.24)	1.21 (1.13, 1.29)	1.20 (1.11, 1.29)	1.15 (1.06, 1.26)	1.10 (1.07, 1.13)
Age difference trend	*P* = 0.03*	*P* = 0.83	*P* = 0.10	*P* = 0.71	*P* = 0.29
≥ 10 years of education	1.17 (0.66, 2.08)	0.92 (0.58, 1.47)	0.53 (0.32, 0.89)	0.87 (0.50, 1.53)	1.12 (0.91, 1.39)
< 10 years of education	1.20 (1.08, 1.34)	1.23 (1.13, 1.35)	1.16 (1.05, 1.28)	1.09 (0.98, 1.22)	1.07 (1.03, 1.11)
Educational difference	*P* = 0.93	*P* = 0.23	*P* < 0.01*	*P* = 0.44	*P* = 0.68
Cold season	1.15 (1.02, 1.30)	1.01 (0.92, 1.11)	0.81 (0.73, 0.90)	0.84 (0.74, 0.96)	0.94 (0.90, 0.99)
Warm season	1.05 (0.90, 1.23)	1.19 (1.03, 1.37)	1.27 (1.10, 1.45)	1.02 (0.86, 1.21)	1.10 (1.01, 1.21)
Seasonal difference	*P* = 0.37	*P* = 0.06	*P* < 0.01*	*P* = 0.08	*P* < 0.01*

*P < 0.05

### Sensitivity analysis results

**Fig. 3:**
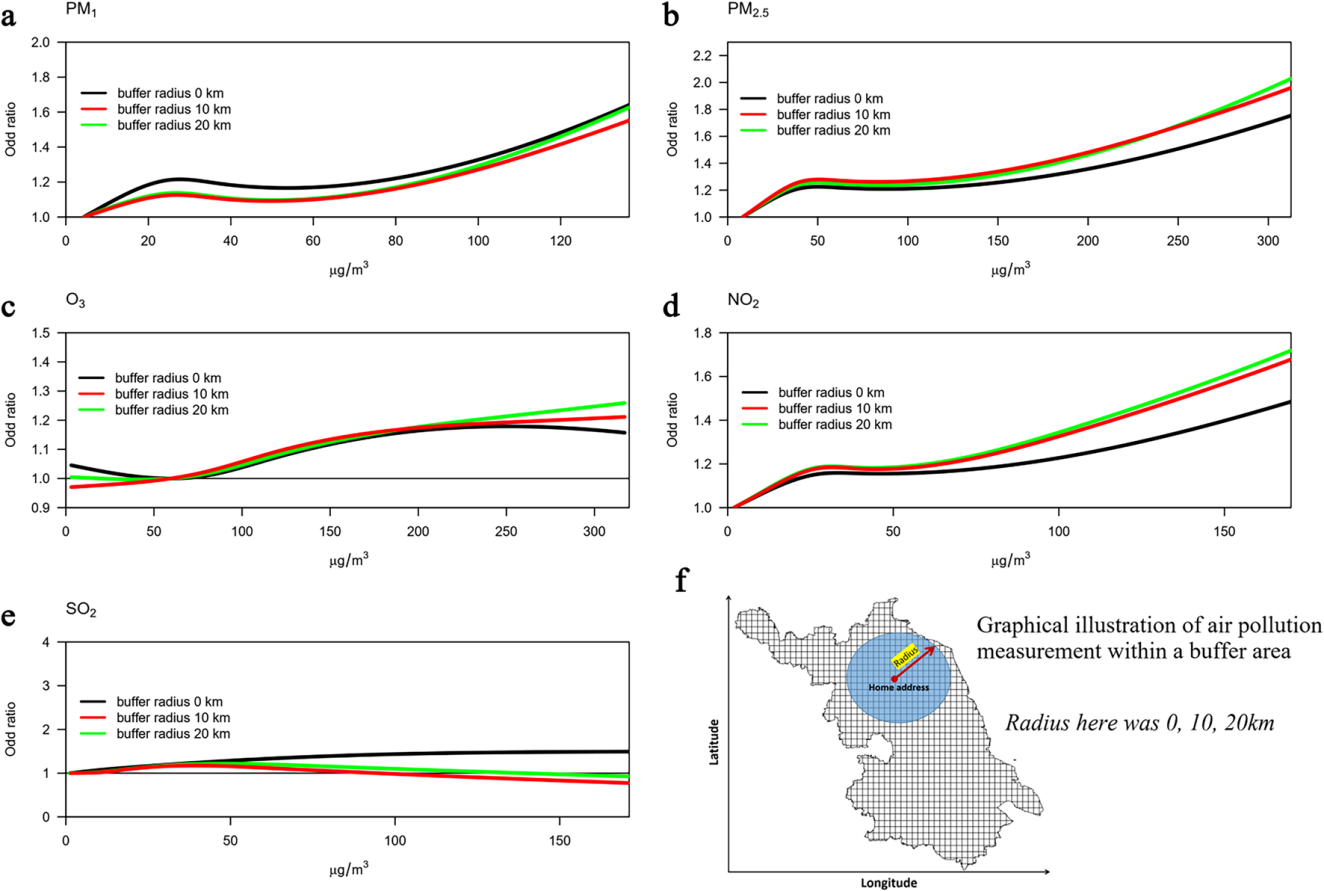
Exposure-response association between exposure to air pollutants and odds of AMI deaths at home using different buffer areas within residential area

For all air pollutants except for SO_2_, the exposure-response associations were robust after considering various buffer areas in measuring residential exposure to air pollution (Fig. 3). Results from the two-pollutant models were consistent with the results from the single-pollutant models (Supplementary Table S2).

## Discussion

This study found that residential exposure to routinely monitored and unmonitored air pollutants (PM_1_, PM_2.5_, NO_2_, SO_2_, or O_3_), even lower than the newest WHO air quality standards, was associated with increased odds of AMI deaths at home. The associations of O_3_ and NO_2_ with odds of AMI deaths at home were stronger in females and in the warm season. The association between PM_1_ and odds of AMI deaths at home appeared to be stronger in young adults than that in the elderly.

Previous studies have provided epidemiological evidence on the association between air pollution exposure and AMI death risk (Liu et al. 2021; Liang et al. 2018; Ren et al. 2010). Air pollution could be from both local sources and neighboring regions. Therefore, linking residential air pollution exposure and AMI deaths at home is essential. The present study is novel because we examined the association between residential air pollution exposure and AMI deaths at home. In many countries (e.g., USA, UK), a large proportion of deaths from cardiovascular diseases (e.g., acute heart attacks) happen at home (Cross and Warraich 2019; Wu et al. 2021). Our results suggested that residential exposure to air pollution may be a trigger of AMI deaths at home. Although Liu et al. have quantified the association between exposure to air pollutants and AMI deaths (Liu et al. 2021), they included all AMI deaths rather than those deaths occurred at home. Therefore, it is challenging to directly compare our results with theirs. Nevertheless, air pollutants such as PM_10_, PM_2.5_, NO_2_, SO_2_, and O_3_ have been shown to be risk factors for AMI mortality, suggesting that residential exposure to these pollutants could increase the risk of AMI deaths in China.

The main strength of this study was that we leveraged high-resolution air pollution data and individual-level health data to examine the association between short-term residential exposure to air pollution and risk of AMI deaths at home. Notably, our study demonstrated that exposure to air pollution lower than the air quality standards recommended by WHO was still associated with a higher risk of AMI deaths. Recently, WHO has released more stringent air quality standards (WHO 2021). The existing studies have consistently reported adverse cardiovascular impacts of low-level air pollution exposure (Liu et al. 2021; Cheng et al. 2021; deSouza et al. 2021). The findings from our study and previous studies suggested that exposure to low-level air pollution is still detrimental to cardiovascular health.

Three main biological mechanisms have been proposed to explain the association between short-term exposure to air pollutants and an increased risk of AMI attacks, including systematic oxidative stress, inflammation, and autonomic imbalance (Claeys et al. 2016). Fine and ultrafine particulate matters (e.g., PM_2.5_) have large surface area and can carry a large number of toxicants (e.g., transition metals) deep into respiratory tract, causing increases in biomarkers (e.g., oxidized low density lipoprotein and interleukin-6) of oxidative stress and inflammatory responses (Claeys et al. 2016; Araujo et al. 2008; Kelly 2003; Lin et al. 2019). Gaseous pollutants such as O_3_ and SO_2_ can modify automatic nervous system balance via the activation of pulmonary neural reflexes, whereby leading to vasoconstriction, vascular congestion, and changes in blood pressure and heart rate (Claeys et al. 2016; Perez et al. 2014). These biological responses promote or accelerate atherothrombosis and could trigger AMI attacks.

We found a stronger association between O_3_ exposure and risk of AMI deaths at home in the warm season than that in the cold season, which was consistent with the finding reported in a previous meta-analysis (Bergmann et al. 2020). This finding could be explained by two reasons. First, ambient O_3_ level is generally higher during warm months (particularly during summer). Second, people tend to open windows or go outdoors more during warm months (Bergmann et al. 2020). We also observed stronger associations of O_3_ and NO_2_ with risk of AMI deaths at home in females, echoing the finding of a previous meta-analysis (Zhao et al. 2017). Clinicians should remind their patients with high risk of AMI attacks (e.g., those with pre-existing cardiovascular diseases, particularly females) to pay attention to air quality forecasting and avoid outdoor activities during days with heavy air pollution.

In the present study, we found an association between short-term exposure to PM_1_ and an increased risk of AMI deaths. PM_1_ has a small particulate size and can deposit deep into lungs, eliciting oxidative stress and inflammation response that can cause damages to heart (Niu et al. 2021). Besides, PM_1_ is able to carry large concentrations of toxic components that can directly pass into circulatory system (Niu et al. 2021; Delfino et al. 2005; Polichetti et al. 2009). The association between PM_1_ and adverse health events (e.g., cardiovascular deaths) has been increasingly reported in China and elsewhere (Chen et al., 2017a, b; Mei et al. 2022; Perez et al. 2009). Available evidence suggested that PM_1_ was responsible for a large proportion of PM_2.5_-related disease burden (Chen et al. 2017a, b; Hu et al. 2018).

We observed a nonlinear exposure-response association between short-term exposure to air pollutants and risk of AMI deaths. Risk of AMI deaths increased faster at lower concentrations of air pollutants and appeared to flatten out at higher concentrations. This finding may be partially explained by the implementation of clean air policy, society-wide health education, and behavior adaptation (Yan et al. 2019). Similar phenomenon has also been reported in other regions of China (Liu et al. 2021; Yan et al. 2019). Adopting a nonlinear assumption of exposure-response association may help identify crucial risk turning point, which is useful for prevention of cardiovascular events (Liu et al. 2021; Yan et al. 2019).

Several limitations of this study should be acknowledged. First, this study was conducted only in one province of China, and cautions are needed when generalizing our findings to other settings. Future studies are warranted to clarify the role of background air pollution level, pollutant composition, and source in assessing the association between air pollution exposure and risk of AMI deaths. Second, we adopted a case-crossover study design that is ideal for controlling for short-term time-invariant cardiovascular risk factors. However, some short-term time-varying factors such as indoor air quality and noise pollution were not taken into account in this study. Third, although we used satellite remote sensing data to estimate residential exposure to air pollution, we could not rule out the possibility that exposure measurement bias may still exist. Fourth, due to the issue of data availability, we were only able to obtain data on AMI patients who died at home but could not obtain death data on AMI patients who died on the way to hospitals and those who died in nursing facilities. We cannot rule out the possibility that the effect of air pollution on AMI mortality may vary by the death place of AMI patients. Fifth, since this was the first study to explore the effect of air pollutants on AMI deaths at home stratified by sex, we were less able to directly compare our findings with previous studies and explore the underlying reasons behind the difference in the association between air pollution exposure and AMI deaths across two sexes. Sixth, only PM_1_, PM_2.5_, NO_2_, SO_2_, and O_3_ were examined in our study, and other air pollutants such as CO (carbon monoxide) that may have an impact on AMI mortality were not included in our study because of the unavailability of high-resolution CO data. Despite the limitations, our findings have potential implications for protecting public health. First, AMI is a major public health issue in China and the associated disease burden shows an upward trend in recent years (Chang et al. 2017; Liu et al. 2017; Li et al. 2022). In China, most AMI deaths take place at home. Second, this study suggests that PM_1_ is an important but currently less reported risk factor of AMI death. Future research is urgently needed to assess the adverse health effect of PM_1_ or finer particulate matter. Third, results of subgroup analyses by season, educational level, age, and sex are useful for targeting vulnerable populations.

## Conclusion

In conclusion, this large-scale case-crossover study in China provides evidence that residential exposure to routinely monitored and unmonitored air pollutants, even at concentrations lower than the newest WHO air quality standards, is still associated with a higher risk of AMI deaths at home. Females are more vulnerable to NO_2_ and O_3_ than males, and young adults are more vulnerable to PM_1_ than elderly. Since air pollution is an important contributor to global cardiovascular disease burden, our findings highlight that continued efforts to lower air pollution or avoid air pollution exposure are necessary in residential settings. Future studies are warranted to understand the biological mechanisms behind the triggering of AMI deaths by air pollution exposure, to develop intervention strategies to reduce AMI deaths triggered by air pollution exposure, and to evaluate the cost-effectiveness, accessibility, and sustainability of these intervention strategies.

## Supplementary Information

ESM 1 (DOCX 59 kb)

## Data Availability

All data generated or analyzed during this study are included in this published article.
