# Supplementary material for: Short-term residential exposure to air pollution and risk of acute myocardial infarction deaths at home in China
Source: Environ Sci Pollut Res Int. 2023 May 29;30(31):76881–90. doi: 10.1007/s11356-023-27813-5 (PMC10300167; doi:10.1007/s11356-023-27813-5)
Supplement: Supplementary file 1 — (DOCX 59 kb) [file 11356_2023_27813_MOESM1_ESM.docx]

**Supplementary materials**

**Short-term residential exposure to air pollution and risk of acute myocardial infarction deaths at home in China**

**Figure S1.** Spatial distribution of acute myocardial infarction deaths at home in Jiangsu Province, China, 2016 to 2019

**Table S1.** Summary statistics of acute myocardial infarction deaths at home in Jiangsu Province from 2016 to 2019

**Table S2.** Association between exposure to air pollutants and odds of acute myocardial infarction deaths in the single- and two-pollutant models


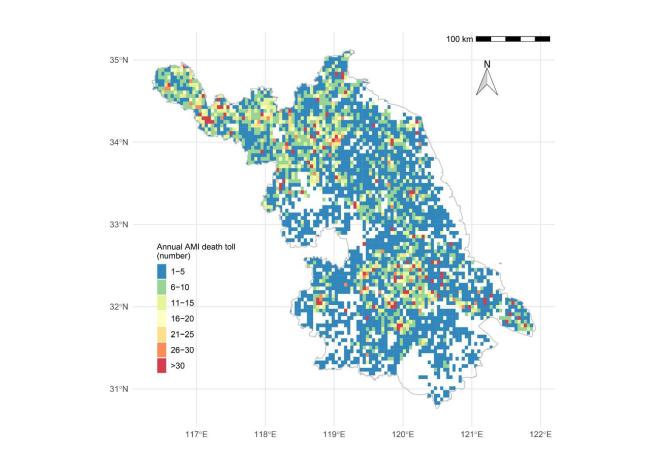


**Figure S1.** Spatial distribution of acute myocardial infarction deaths at home in Jiangsu Province, China, 2016 to 2019

**Table S1.** Summary statistics of acute myocardial infarction (AMI) deaths at home in Jiangsu Province from 1 January 2016 to 31 December

2019

| Variable | Number | Percentage (%) |
| --- | --- | --- |
| **All AMI deaths at home** | 102, 183 | 100 |
| **Sex** |  |  |
| Males | 50,352 | 49 3 |
| Females | 51,831 | 50.7 |
| **Age at death (years)** |  |  |
| ≤64 | 14,534 | 14 2 |
| 65-74 | 16,202 | 15.9 |
| 75-84 | 34,041 | 33.3 |
| ≥ 85 | 37,406 | 36 6 |
| **Educational attainment** |  |  |
| ≥ 10 years | 99, 106 | 97 0 |
| < 10 years | 3,077 | 3.0 |
| **Season** |  |  |
| Warm season (April to September) | 43,656 | 42 7 |
| Cold season (January to March, October to December) | 73,722 | 57.3 |

**Table S2**. Association between exposure to air pollutants and odds of acute myocardial infarction deaths in the single- and two-pollutant models.

| **Models** | **Pollutants** | **Lags** | **Odds ratio & 95% confidence interval** | | |
| --- | --- | --- | --- | --- | --- |
|  |  |  | **IQR increase** | **China’s air quality criteria** | **WHO’s air quality criteria** |
| Single-pollutant | PM_1_ | Lag0-1 | 1.20 (1.08, 1.33) | NA | NA |
| Two-pollutant | PM_1_+NO_2_ | Lag0-1 | 1.20 (1.08, 1.33) | NA | NA |
| Two-pollutant | PM_1_+SO_2_ | Lag0-1 | 1.20 (1.08, 1.34) | NA | NA |
| Two-pollutant | PM_1_+O_3_ | Lag0-1 | 1.17 (1.05, 1.30) | NA | NA |
| Single-pollutant | PM_2.5_ | Lag0-1 | 1.22 (1.12, 1.33) | 1.21 (1.11, 1.32) | 1.05 (1.03, 1.08) |
| Two-pollutant | PM_2.5_+NO_2_ | Lag0-1 | 1.21 (1.11, 1.33) | 1.20 (1.10, 1.31) | 1.05 (1.03, 1.08) |
| Two-pollutant | PM_2.5_+SO_2_ | Lag0-1 | 1.22 (1.12, 1.33) | 1.20 (1.10, 1.31) | 1.06 (1.03, 1.08) |
| Two-pollutant | PM_2.5_+O_3_ | Lag0-1 | 1.18 (1.08, 1.29) | 1.17 (1.07, 1.28) | 1.05 (1.02, 1.07) |
| Single-pollutant | NO_2_ | Lag0-2 | 1.13 (1.03, 1.25) | 1.19 (1.05, 1.33) | 1.15 (1.03, 1.28) |
| Two-pollutant | NO_2_+PM_1_ | Lag0-2 | 1.12 (1.01, 1.23) | 1.17 (1.03, 1.31) | 1.13 (1.02, 1.26) |
| Two-pollutant | NO_2_+PM_2.5_ | Lag0-2 | 1.11 (1.00, 1.22) | 1.14 (1.01, 1.28) | 1.12 (1.01, 1.25) |
| Two-pollutant | NO_2_+SO_2_ | Lag0-2 | 1.11 (1.00, 1.22) | 1.08 (0.93, 1.24) | 1.12 (1.00, 1.25) |
| Two-pollutant | NO_2_+O_3_ | Lag0-2 | 1.03 (0.93, 1.14) | 1.06 (0.93, 1.20) | 1.03 (0.92, 1.16) |
| Single-pollutant | SO_2_ | Lag0-5 | 1.14 (1.02, 1.27) | 1.49 (0.41, 5.37) | 1.24 (1.10, 1.40) |
| Two-pollutant | SO_2_+PM_1_ | Lag0-5 | 1.12 (1.01, 1.25) | 1.17 (0.32, 4.29) | 1.22 (1.08, 1.37) |
| Two-pollutant | SO_2_+PM_2.5_ | Lag0-5 | 1.09 (0.98, 1.22) | 1.06 (0.29, 3.92) | 1.17 (1.04, 1.33) |
| Two-pollutant | SO_2_+NO_2_ | Lag0-5 | 1.24 (1.08, 1.41) | 1.17 (0.32, 4.28) | 1.42 (1.22, 1.67) |
| Two-pollutant | SO_2_+O_3_ | Lag0-5 | 1.02 (0.90 1.15) | 1.54 (0.43, 5.56) | 1.11 (0.97, 1.26) |
| Single-pollutant | O_3_ | Lag0-5 | 1.07 (1.03, 1.12) | 1.13 (1.08, 1.18) | 1.04 (1.01, 1.07) |
| Two-pollutant | O_3_+PM_1_ | Lag0-5 | 1.08 (1.04, 1.12) | 1.13 (1.07, 1.18) | 1.04 (1.01, 1.07) |
| Two-pollutant | O_3_+PM_2.5_ | Lag0-5 | 1.07 (1.03, 1.11) | 1.11 (1.06, 1.17) | 1.04 (1.01, 1.07) |
| Two-pollutant | O_3_+NO_2_ | Lag0-5 | 1.08 (1.03, 1.12) | 1.13 (1.07, 1.19) | 1.04 (1.01, 1.07) |
| Two-pollutant | O_3_+SO_2_ | Lag0-5 | 1.06 (1.02, 1.11) | 1.11 (1.05 1.17) | 1.03 (1.00 1.06) |

IQR is inter-quartile range; Air quality standards for PM_2.5_ are 75μg/m^3^ (China) and 15μg/m^3^ (WHO), for NO_2_ are 80μg/m^3^ (China) and 25μg/m^3^ (WHO), for SO_2_ are 150μg/m^3^ (China) and 40μg/m^3^ (WHO), for O_3_ are 160μg/m^3^ (China) and 100μg/m^3^ (WHO); NA means no existing air quality standard for that air pollutant.
